# Heterogating Gel Iontronics: A Revolution in Biointerfaces and Ion Signal Transmission

**DOI:** 10.3390/gels10090594

**Published:** 2024-09-15

**Authors:** Zhixin Wu, Ziguang Zhao

**Affiliations:** School of Future Technology, University of Chinese Academy of Sciences, Beijing 100190, China; wuzhixin23@mails.ucas.ac.cn

**Keywords:** iontronics, heterogels, ion transmission

## Abstract

Currently, existing iontronic systems are limited and struggle to process electronic-to-multi-ionic transport, resulting in interchange inefficiencies and incompatibilities between artificial ion devices and biological tissue interfaces. The development of heterogating gel iontronics offers a significant advancement in bridging this gap, drawing inspiration from the complex ionic transmission mechanisms found in biological synapses within neural networks. These heterogating gels utilize a biphasic architecture, where the heterointerface effect constructs ionic transfer energy barriers, enabling distinct signal transmission among different ions. In systems with multiple ion species, heterogating gel iontronics allow for precise control of ion transmission, realizing hierarchical and selective cross-stage signal transmission as a neuromorphic function. This perspective highlights the vast potential of heterogating iontronics in applications such as biosensing, neuroprosthetics, and ion separation technologies. Meanwhile, it also addresses the current challenges, including scaling production, ensuring biocompatibility, and integrating with existing technologies, which are crucial for future development. The advancement of heterogating gels is expected to promote the integration between abiotic and biotic systems, with broad implications for smart sensors, bioneural devices, and beyond.

## 1. The Complexity of Biological Neural Networks

**Figure 1:**
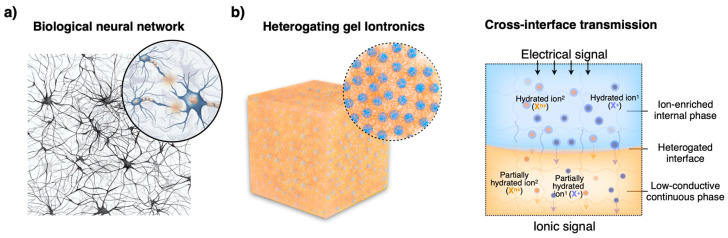
The internal structure of biological neural networks (**a**) and heterogating gel iontronics (**b**). The phase separation interface can act as a functional gating that facilitates selective ion transmission. (Note: Numbers represent different ions.)

Biological neural networks exemplify nature’s intricacy, where the precise and diverse transmission of ionic signals is central to life’s most complex processes [1,2,3]. Between interconnected neurons, synaptic interfaces maintain a delicate balance of ionic and neurotransmitter transmission, facilitating the diverse communication of biological signals [4] (Figure 1a). The synapse is a sophisticated structure where ions and neurotransmitters are released and received, enabling the propagation of nerve impulses that regulate everything from muscle contractions to cognitive functions [5,6].

At the core of such signaling processes are ion channels and transporters that selectively allow ions like sodium, potassium, calcium, and chloride to flow in and out of neurons, generating actuation potentials that traverse and regulate the neural network. Selective ion transport, as a dynamic complex process, is tightly controlled by the neuron’s membrane potential and the specific ionic environment [7,8]. The synaptic interface possesses the capability for rapid and precise adjustments to ensure the accurate transmission of ionic signals. Replicating this intricate ion-based mechanism in artificial systems remains a significant challenge, with key considerations including the construction of neuromorphic ion transmission pathways and effective interaction with biological interfaces [9].

## 2. Current Challenges in Ionic and Electronic Material Devices

Despite the significant advancements in smart materials and devices, previous electronics and iontronics still exhibit inherent limitations in signal transmission when interfacing with biological systems. Traditional electronic devices primarily rely on electrical impulse signals to interact with biological tissue [10,11]. Iontronic devices can bridge the gap between electronic signals and biological ionic signals, showing promise by achieving the translation of electronic inputs into ionic outputs [12,13]. However, compared to bioneuronal networks with diverse ionic transmission, existing iontronic systems are still limited, typically handling only single ion species and lacking the ability to selectively control the transport of different ions. For instance, current hydrogel-based iontronics fail to demonstrate distinct ion transmission, reducing their effectiveness in applications that require precise biosignal modulation for biological tissues [14,15]. This gap between current device capabilities and biological requirements highlights the need for new materials and devices with an effective gating mechanism that more accurately replicates the intricate ion transport mechanisms inherent to biological systems.

## 3. Innovative Design and Functions of Heterogating Gel Iontronics

To address these challenges, the development of heterogating gel iontronics represents a significant breakthrough [16]. Heteromaterials draw inspiration from complex neural networks, particularly the highly ion-selective synapse interfaces found in biological systems. The design of multiple heterogating interfaces involves a biphasic architecture, consisting of an ion-enriched internal gel phase (IE phase) and a low-conductivity continuous gel phase (LC phase). An in-situ interface polymerization strategy was introduced to fabricate such heteromaterials [17,18]. Within these materials, the phase separation is not just a structural feature but a functional innovation that facilitates selective ionic cross-interface transport. The heterogating interfaces between these two phases can act as cascaded, selective gates that regulate ion movement based on the ions’ intrinsic properties, such as size, charge, and hydration energy. Such interfacial mechanisms effectively mimic the ion selectivity of multiple synapse interfaces within bioneural networks, creating ionic cross-medium energy transfer barriers that allow for precise control of ion transport (Figure 1b). By tuning the properties of these heterogating interfaces, the system can differentiate between multiple ion species and selectively transmit ion signals. This capability to manage multi-ionic transmission with high specificity makes heterogating gels a revolutionary advancement in the field of ion systems, offering a promising solution to the limitations of current iontronic materials and devices.

**Figure 2:**
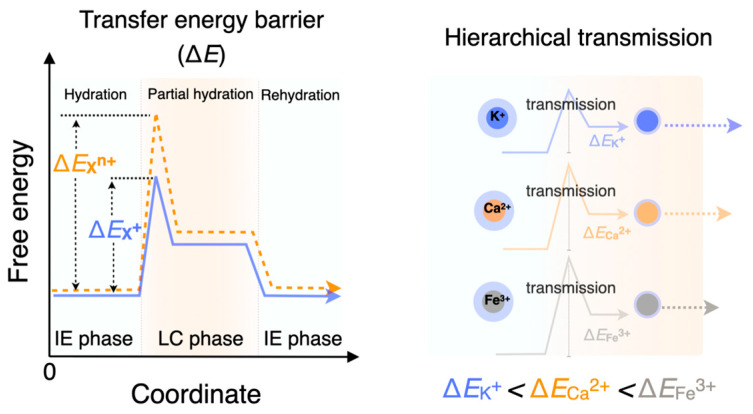
The distinction of the transfer energy barrier between different ions, accompanied by the switching between hydration and partial hydration states across the heterogeneous interfaces, determines the prioritization and hierarchy of multi-ion transmission.

The mechanism by which heterogating gels achieve selective ion transport is derived from their phase-separated heterogeneous structure. When an electrical signal is applied, ions within the IE phase have to partially shed their hydration shells to cross the heterointerface into the LC phase. During ionic cross-interface transmission, partial shedding and reconfiguration of hydration shells, corresponding to the ionic partial hydration and rehydration states, can occur alternately and successively. Within the cascaded heterogating interface, the hydration–dehydration energies between different ions play a crucial role in determining how ions experience the intrinsic transfer energy barrier, thereby dominating the distinct ionic signal transmissions of iontronics (Figure 2). For example, ions with higher charge densities, such as Ca^2+^ and Fe^3+^, face greater energy requirements to shed their hydration shells, encountering higher energy barriers at the heterointerface. Conversely, ions like K^+^, with lower charge densities and smaller hydration shells, pass through more easily. This selective transport can be finely controlled by external electrical signals, which regulate the timing and sequence of ion transmission, including hierarchical and selective cross-stage signal transmission. Heterogating gels function as an ion transport system that not only differentiates between ion species but also prioritizes their transmission based on the specific demands of the application. The comparative analysis table summarizes and contrasts signal transmission features of state-of-the-art iontronic systems, which would further exhibit the important functions (i.e., signal storage/selectivity/multi-species signal hierarchy) derived from the heterogating gel iontronics (Table S1). This unprecedented level of control over ion transport marks a significant advancement in the development of devices capable of interfacing with biological tissues.

Heterogating gel iontronics, with their ability to facilitate electronic-to-multi-ionic signal transmission, hold significant promise for applications in biosensing and energy storage. As research in this field progresses, we anticipate the emergence of increasingly sophisticated devices capable of seamlessly integrating with biological systems. These ion devices could interface with complex biological interfaces, offering new insights into their signal transmission functions and paving the way for the development of advanced smart abiotic–biotic systems.

A particularly promising area of future research lies in the integration of ion-selective piezoelectric and piezoresistive properties into heterogating gels. These advanced materials could enable the creation of devices that not only control ion transport with high specificity but also respond dynamically to mechanical stimuli. Based on piezoelectric effects, such devices could generate diverse ion-to-electrical signals in response to mechanical deformation, making them ideal for applications where mechanical and ionic interactions are critical, such as in wearable sensors or implantable devices. Their piezoresistive properties could further enhance the functionality of these gels by enabling real-time monitoring of mechanical changes within biological tissues, providing valuable feedback for both diagnostic and therapeutic purposes. Otherwise, combining heterogating gels with micro-nano fabrication technologies, such as 3D printing, provides a robust platform for developing next generation iontronic devices with improved functionality and design flexibility. By utilizing the precision and customization capabilities of 3D printing, heterogating gels can be structured into complex, multi-scale architectures that optimize ion transport efficiency.

**Figure 3:**
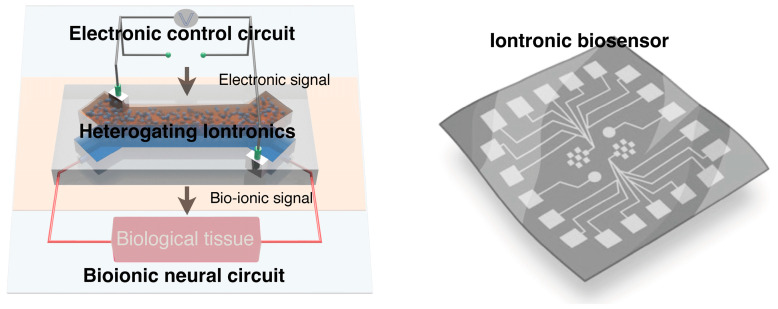
Heterogating iontronics with electronic-to-multi-ionic signal transmission demonstrate promising biosensing applications for abiotic–biotic systems.

Heterogating gels with excellent biocompatibility could also play a crucial role in the development of artificial biohybrid systems by offering precise control over ion transport, ensuring compatibility and functionality (Figure 3). This could lead to the creation of new types of implants, such as artificial organs or bioelectronic devices, that work harmoniously with the body’s natural processes. In neuroprosthetics, heterogating gels could be used to establish more natural and responsive connections between ion signals and the nervous system, allowing for more precise control of movement and enhanced overall functionality. By integrating heterogating gels with existing neural electrode technology, it would enhance compatibility between artificial systems and neural tissues, paving the way for more seamless, stable, and durable neuroprosthetic implants. Heterogating gels have the potential to revolutionize the fields of biosensing and brain-machine interfaces. Their ability to selectively detect and respond to changes in ionic concentrations in biological systems could lead to the development of highly sensitive diagnostic tools capable of early disease detection and real-time health monitoring. Furthermore, heterogating gel iontronics could contribute to tissue regeneration by precisely modulating the ionic environment around cells through their advanced ionic signal transmission and gating functionalities, which are critical for directing cellular behaviors.

Moreover, heterogating gels hold great potential in the field of ion separation technology. The ability of these gel materials to selectively control the transport of different ions based on their size, charge, and hydration energy can be leveraged to develop advanced ion sieving systems. For example, heterogating materials can be integrated into reverse osmosis technology. Such systems could be critical in applications ranging from water purification and desalination to the extraction of valuable metals in industrial processes. Additionally, the principles underlying heterogating gels could be applied to energy storage systems, where selective ion transport is essential for improving the efficiency and performance of batteries and fuel cells. By controlling the movement of specific ions, it is possible to enhance the energy density and stability of these systems, making them more effective and reliable for long-term use.

## 4. Conclusions

The development of heterogating gel iontronics represents a significant leap forward in bridging the gap between electronic and biological systems. Inspired by the heterointerfacial signal-gating mechanisms found in biological neural networks, these biphasic-gel iontronic systems offer a novel approach to diverse electronic-ionic signal transmission. By utilizing phase-separated heteronetwork structures, which integrate an ion-enriched internal gel phase with a low-conductivity continuous gel phase, these systems establish ion transfer energy barriers that are precisely controlled by the hydration-dehydration energies of different ions. This results in a highly selective and tunable ion transport mechanism that mimics the hierarchical signal transmission of neuromorphic systems. In the future, by incorporating dynamic covalent bonds, hydrogen bonds, and other reversible cross-linking structures into the heterogating gel network, self-healing properties will also be realized to enhance practical application capabilities.

However, several challenges must be addressed to realize the full potential of heterogating gels, particularly their scalability for industrial production and the reproducibility of their selective ion transport properties. Ensuring the long-term stability and biocompatibility of these materials in biological environments is crucial for their application in medical devices. Another significant challenge is integrating heterogating gels with existing technologies. While these materials offer substantial advantages in terms of selectivity and control over ion transport, their compatibility with the current infrastructure of electronic and iontronic devices is essential.

Despite the challenges, heterogating iontronics hold the potential to drive groundbreaking innovations, effectively bridging the gap between electronic systems and living tissues. These material systems are expected to offer new possibilities for technological development and interdisciplinary exploration. As research advances, the further integration of ion gating and iontronic functions promises unprecedented advancements in biosensing, neuroprosthetics, and neuromorphic devices.

## Data Availability

No new data were created or analyzed in this study.

## Supplementary Materials

The following supporting information can be downloaded at: https://www.mdpi.com/article/10.3390/gels10090594/s1, Table S1: Signal transmission features of previous iontronic systems.
