# Supplementary material for: Heterogating Gel Iontronics: A Revolution in Biointerfaces and Ion Signal Transmission
Source: Gels. 2024 Sep 15;10(9):594. doi: 10.3390/gels10090594 (PMC11431666; doi:10.3390/gels10090594)
Supplement: Supplementary file 1 [file gels-10-00594-s001.zip › gels-3200574-supplementary.pdf]

gels MDPI

Perspective

Heterogating Gel Iontronics: A Revolution in Biointerfaces and Ion Signal Transmission

Zhixin Wu[1] and Ziguang Zhao[1,*]

[1] School of Future Technology, University of Chinese Academy of Sciences, Beijing 100190, P. R. China, wuzhixin23@mails.ucas.ac.cn (W.Z.); zhaoziguang@ucas.ac.cn (Z.Z.)
* Correspondence: zhaoziguang@ucas.ac.cn (Z.Z.)

This PDF file includes:

Tables S1

References

Table S1. Signal transmission features of previous iontronic systems.

Iontronics	Gating	Stimulus signal attribute	Transmitted signal attribute	Ion signal storage	Ionic signal selectivity	Hierarchical multi-ionic signals
Metal-based systems	Voltage gating [1-2]	Electricity	Single ion	Yes	No	No
Metal oxide-based systems	Voltage gating [3-5]	Electricity	Single ion	Yes	No	No
Silicon-based systems	Non-gating [6-8]	Electricity	Single ion	Yes	No	No
	Voltage gating [9-10]	Electricity	Single ion or multiple ions	Yes	No	No
Conjugated polymer-based systems	Non-gating [11-12]	Electricity	Single ion	Yes	No	No
	Voltage gating [13-16]	Electricity	Single ion	Yes	No	No
	Ligand gating [17]	Ions or polyatomic ions	Multiple ions	Yes	Yes	No
Ionic liquid-based systems	Non gating [18-19]	Electricity	Single ion	Yes	No	No
	Voltage gating [20-21]	Electricity	Single ion or multiple ions	Yes	No	No
Liquid-based systems	Non-gating [22]	Electricity	Single ion	Yes	No	No
	Optical gating [23]	Light	Single ion	Yes	No	No
	Voltage gating [24]	Electricity	Single ion	Yes	No	No
Gel-based systems	Non-gating [25-27]	Electricity/ Mechanical force	Single ion	Yes	No	No
	Voltage gating [28]	Electricity	Single ion	Yes	No	No
	Piezopotential gating [29]	Piezopotential	Single ion	Yes	No	No
Gel-based junctions	Voltage gating [30-31]	Electricity	Single ion	Yes	No	No
Nanocomposite-based systems	Non-gating [32]	Electricity	Single ion	Yes	No	No
	Voltage gating [33-34]	Electricity	Single ion	Yes	No	No
Nanofluidics-based systems	pH- / temperature-gating [35-37]	pH/ temperature	Single ion	No	No	No
	Light gating [38]	Light	Single ion	No	No	No
	Interaction gating [39]	Ligand	Single ion	No	Yes	No
	Voltage gating [40-41]	Electricity	Single ion	Yes	No	No
**HBG-based systems**	**Cascade heterogating/ Cascade chemical-heterogating**	Electricity	**Multiple ions**	**Yes**	**Yes**	**Yes**
